# Synthesis and Antimalarial Activity of Novel Dihydro-Artemisinin Derivatives

**DOI:** 10.3390/molecules16064527

**Published:** 2011-05-30

**Authors:** Yang Liu, Kunqiang Cui, Weiqiang Lu, Wei Luo, Jian Wang, Jin Huang, Chun Guo

**Affiliations:** 1 Key Laboratory of Structure-Based Drug Design & Discovery of Ministry of Education, School of Pharmaceutical Engineering, Shenyang Pharmaceutical University, Shenyang 110016, China; E-Mails: syphuly0325@yahoo.com.cn (Y.L.); l198278w@yahoo.com.cn (W.L.); wjmed@163.com (J.W.); 2 School of Pharmacy, East China University of Science and Technology, Shanghai 200237, China; E-Mails: cscs12683@126.com (K.C.); luweiqiangnb@126.com (W.L.); huangjin@ecust.edu.cn (J.H.)

**Keywords:** falcipain-2 inhibitors, dihydroartemisinin derivatives, inhibition activity, molecular docking studies, SARs

## Abstract

The *Plasmodium falciparum* cysteine protease falcipain-2, one of the most promising targets for antimalarial drug design, plays a key role in parasite survival as a major peptide hydrolase within the hemoglobin degradation pathway. In this work, a series of novel dihydroartemisinin derivatives based on (thio)semicarbazone scaffold were designed and synthesized as potential falcipain-2 inhibitors. The *in vitro* biological assay indicated that most of the target compounds showed excellent inhibition activity against *P. falciparum* falcipain-2, with IC_50_ values in the 0.29–10.63 μM range. Molecular docking studies were performed to investigate the binding affinities and interaction modes for the inhibitors. The preliminary SARs were summarized and could serve as a foundation for further investigation in the development of antimalarial drugs.

## 1. Introduction

**Figure 1:**
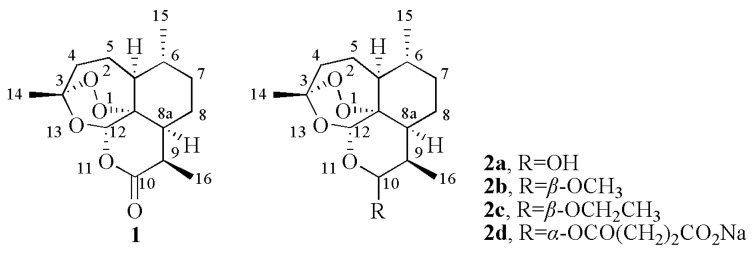
Structures of artemisinin and its derivatives.

Malaria is the most common parasitic disease in the World. *Plasmodium falciparum* is the major cause of sever malaria and death and has developed resistance to most available antimalarial drugs [1]. Artemisinin (**1**), isolated from the *Artemisia annua* L., and its derivatives **2a-d** (Figure 1) are effective antimalarial drugs against multidrug-resistant *P. falciparum* [2]. The WHO has recommended artemisinin-based combination therapies (ACTs) as first-line treatment for uncomplicated *P. falciparum* malarial since 2001 [3]. However, as *P. falciparum* resistance to artemisinin has emerged [4], development of antimalarial drugs against new targets is an urgent priority.

Among the promising new targets, *P. falciparum* cysteine protease falcipain-2, which plays a key role for the parasite survival as a major peptide hydrolase within the hemoglobin degradation pathway, is one of the most attractive targets for antimalarial drug design [5]. Falcipain-2 inhibitors can block parasite protein biosynthesis by preventing host hemoglobin hydrolysis [6]. Therefore, discovery of new falcipain-2 inhibitors with acceptable properties is a meaningful work.

Iron is essential for the biological activities of some plasmodial proteins and due to the metal chelating properties, the (thio)semicarbazone moiety has shown activity as a potential facipain-2 inhibitor aimed at preventing the growth of malarial parasites [7,8,9].

In this work, a series of novel dihydroartemisinin derivatives **10a-l** and **12a-f** were designed and synthesized by incorporating the above two biologically active scaffolds, dihydroartemisinin and (thio)semicarbazone. The *in vitro* biological assay was carried out against *P. falciparum* falcipain-2. The preliminary SARs were summarized and could benefit to the subsequent drug design process.

## 2. Results and Discussion

### 2.1. Chemistry

**Scheme 1:**
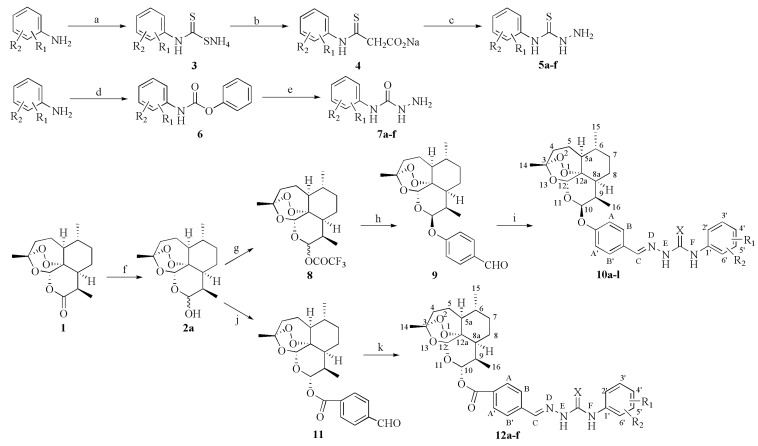
The synthetic routes for compounds **10a-l** and **12a-f**.

The target compounds **10a-l** and **12a-f** were synthesized through a general condensation procedure of 4-substituted benzaldehydes **9** and **11** with thiosemicarbazides **5a-f** or semicarbazides **7a-f** respectively (Scheme 1).

Compounds **9** and **11** were the key intermediates of the synthetic process. Compound **9** was synthesized by an indirect method rather than the generally direct etherification catalyzed by BF_3_⋅Et_2_O [10]. Artemisinin (**1**) was reduced with NaBH_4_ based on an effective route to dihydroartemisinin (**2a**) [11] which was activated by trifluoroacetic anhydride (TFAA) in the presence of Et_3_N to form trifluoroacetate **8**. Without separation, compound **8** reacted with 4-hydroxybenzaldehyde to give compound **9** which was purified on a silica gel column. Compound **11** was synthesized directly through the esterification of dihydroartemisinin **2a** and 4-formylbenzoic acid catalyzed by dicyclohexylcarbodiimide (DCC) and 4-*N*,*N*-dimethylaminopyridine (DMAP) and was also purified on a silica gel column [12]. Thiosemicarbazides **5a-f** were obtained from different anilines by a literature method [13]. The anilines reacted with carbon disulphide in the presence of concentrated ammonia to yield dithiocarbamates **3**. These compounds on treatment with sodium monochloroacetate followed by condensation with hydrazine hydrate gave **5a-f**. Semicarbazides **7a-f** were also obtained from anilines through acylation with phenyl chloroformate to give carbamates **6**, which were condensed with hydrazine hydrate to yield **7a-f** [14]. 

All the target compounds were identified as single isomers due to the configuration of compounds **9** and **11** confirmed by ^1^H-NMR. The configuration at C-10 was assigned based on the coupling constant between H-9 and H-10. A small coupling constant (*J* = 3.6 Hz) was observed in *β*-arteether, while a large coupling constant (*J* = 9.2 Hz) was observed in *α*-arteether [11] and a similar rule was found in dihydroartemisinin aromatic ethers in other reports [15,16]. In compounds **9** and **11**, these coupling constants were 3.3 Hz and 9.6 Hz, respectively, which indicated the former was a *β*-isomer and the latter was an *α*-isomer.

### 2.2. Biological Assay in Vitro

The *in vitro P. falciparum* falcipain-2 inhibition assay against was carried out according to a previously reported method by taking *N*-(L-3-*trans*-carboxyoxiran-2-carbonyl)-L-leucyl)-amido(4-guanido)butane (**E-64**) as a positive control and DMSO as a negative control [17]. Falcipain-2 could hydrolyse the fluorogenic substrate benzyloxycarbonyl-Leu-Arg-7-amino-4-methylcoumarin (*Z*-Leu-Arg-AMC) to produce coumarin and its activity could be evaluated by monitoring the fluorescence of the coumarin. Activity of all the tested compounds is shown in Table 1.

All the tested compounds were found to be inhibitors of falcipain-2 with inhibition rates greater than 20% at 10 μM concentration level and IC_50_ values ranging from 0.29 to 10.63 μM. The preliminary SARs showed that the ester linker in compounds **12a-f** was more effective than the ether linkers in compounds **10a-l** with the same substitution on the aromatic ring (e.g., **12a** and **10a**) and the latter displayed equivalent efficacy between semicarbazones and thiosemicarbazones (e.g., **10e** and **10k**). Furthermore, in the same kind of scaffolds, the introduction of fluoro or ethoxyl groups at the C-4 position as monosubstituents on the aromatic ring could improve the activity (e.g., **12b** and **10c**) and among disubstituted compounds, the ones with methyl group disubstitution at the C-3 and C-5 positions on the aromatic ring presented better activities (e.g., **10k** and **12e**). These implications would benefit a drug design in future research.

### 2.3. Molecular Docking Studies

**Figure 2:**
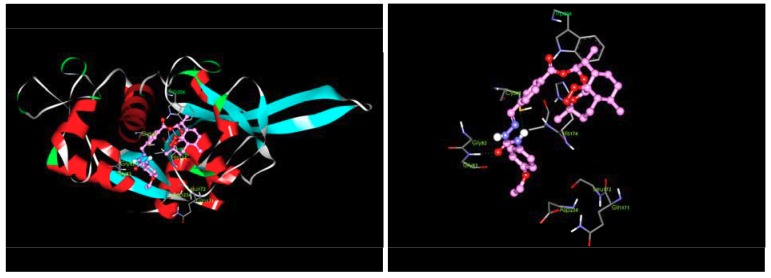
Proposed binding mode of compound **12c** in the active site of falcipain-2.

Molecular docking studies were performed to investigate the binding affinities and interaction modes for the inhibitors using AutoDock 3.05 [18]. As illustrated in Figure 2, residues Trp206, Cys42, His174, Gly82, Gly83, Leu172, Asp234 and Gln171 form a pocket for ligand binding, and the most potent compound **12c** forms hydrogen bonds with Cys42 and Gly83.

## 3. Experimental 

### 3.1. Instruments and Reagents

Melting points were taken on glass slides with X-4 digital display microscopic melting point apparatus and were presented uncorrected. ^1^H-NMR and ^13^C-NMR spectra were recorded using a Bruker ARX-300 spectrometer with CDCl_3_ as solvent and TMS as internal standard. Electrospray ionization mass spectra (ESIMS) were recorded on a Waters Quattro micro API. All reagents were commercially available and were used with purification as needed. Chromatography was carried on silica gel (200–300 mesh). All reactions were monitored by TLC (silica gel plates with fluorescence F_254_ were used).

### 3.2. General Procedure for the Synthesis of N^4^-(Substitued Phenyl)thiosemicarbazides ***5a-f***

To an EtOH solution (50 mL) of aniline (0.1 mol), concentrated ammonia (20 mL) was slowly added. The mixture was cooled below 20 °C and CS_2_ (8 mL) was added dropwise during a period of 15 min. After 1 h, sodium monochloroacetate (14 g, 0.12 mol) was added and vigorously stirred for 30 min followed by adding hydrazine hydrate (80%, 12.5 mL). After 1 h, the mixture was cooled overnight and the crude reaction mixture was filtered and recrystallized from EtOH.

### 3.3. General Procedure for the Synthesis of N^4^-(Substitued Phenyl)semicarbazides ***7a-f***

To a mixture of aniline (0.05 mol), pyridine (5 mL) and CH_2_Cl_2_ (30 mL), phenyl chloroformate (6.3 mL, 0.05 mol) was added dropwise under ice-water bath and reacted at room temperature for 4 h. The mixture was then evaporated under reduced pressure and the residue was poured into saturated NaCl solution for salting out. The precipitate was filtered and dried followed by refluxing in hydrazine hydrate (80%, 10 mL) for 12 h. After cooling, the crude reaction mixture was filtered and recrystallized from EtOH.

### 3.4. General Procedure for the Synthesis of Dihydroartemisinin *(**2a**)*

To a stirred solution of artemisinin (**1**, 2 g, 7.08 mmol) in CH_3_OH, NaBH_4_ (0.40 g, 10.62 mmol) was added at 0–5 °C over a period of 20 min. After being stirred for an additional 3 h under the same condition, the mixture was neutralized with glacial acetic acid while the temperature was maintained at 0–5 °C, concentrated by evaporating most CH_3_OH, diluted with cold water (100 mL) and stirred for 15 min at room temperature. The precipitate was collected, washed with water (100 mL × 3) and dried. Yield 97.15%, m.p. 143–145 °C.

### 3.5. General Procedure for the Synthesis of 4-[(10S)-Dihydroartemisinin-10-oxy]benzaldehyde N^4^-(substitued phenyl)(thio)semicarbazones ***10a-l***

To a CH_2_Cl_2_ solution (50 mL) of dihydroartemisinin (**2a**, 11.36 g, 40 mmol) and triethylamine (11.08 mL, 80 mmol), TFAA (11.12 mL, 80 mmol) in CH_2_Cl_2_ (30 mL) was added dropwise at −5–0 °C. After 1 d, 4-hydroxylbenzaldehyde (9.76 g, 80 mmol) was added and stirred for 12 h at the same temperature. The reaction solution was quenched with saturated NaHCO_3_ solution and washed by saturated NaHCO_3_ solution (50 mL × 5) and water (50 mL × 5), respectively. The organic layer was dried (Na_2_SO_4_) and concentrated in vacuo. The residue was purified on silica gel column with petroleum ether/ethyl acetate (8:1) to afford **9**. Yield 40.29%, m.p. 88–90 °C. MS (ESI) *m/z*: 411 (M+Na); ^1^H-NMR (300 MHz, CDCl_3_) *δ*: 0.97 (3H, d, *J* = 6.0 Hz, 16-CH_3_), 1.03 (3H, d, *J* = 7.2 Hz, 15-CH_3_), 1.45 (3H, s, 14-CH_3_), 2.34–2.44 (1H, m, 4-H), 2.83–2.88 (1H, m, 9-H), 5.44 (1H, s, 12-H), 5.62 (1H, d, *J* = 3.3 Hz, 10-H), 7.23 (2H, d, *J* = 8.7 Hz, AA’-2H), 7.84 (2H, d, *J* = 8.7 Hz, BB’-2H), 9.90 (1H, s, C-H); ^13^C-NMR (75 MHz, CDCl_3_) *δ*: 191.17, 162.65, 132.20, 131.03, 116.95, 104.63, 100.40, 88.65, 81.11, 52.74, 44.52, 37.69, 36.57, 34.85, 31.10, 26.29, 24.88, 24.67, 20.56, 13.13. Equimolar quantities of **9** (2 mmol) and **5a-f** (2 mmol) or **7a-f** (2 mmol) was dissolved in EtOH (20 mL) catalyzed by glacial acetic acid (3 drops) and stirred for 2 h at room temperature. The precipitate was filtered and purified on silica gel column with petroleum ether/ethyl acetate (3:1) to afford **10a-f** or with CH_2_Cl_2_/CH_3_OH (80:1) to afford **10g-l**.

*4-[(10S)-Dihydroartemisinin-10-oxy]benzaldehyde N^4^-(2-fluorophenyl)thiosemicarbazone* (**10a**). Yield 19.90%, m.p. 108–109 °C. MS (ESI) *m/z*: 556 (M+H); ^1^H-NMR (300 MHz, CDCl_3_) *δ*: 0.97 (3H, d, *J* = 6.0 Hz, 16-CH_3_), 1.03 (3H, d, *J* = 7.2 Hz, 15-CH_3_), 1.45 (3H, s, 14-CH_3_), 2.34–2.44 (1H, m, 4-H), 2.82–2.87 (1H, m, 9-H), 5.47 (1H, s, 12-H), 5.57 (1H, d, *J* = 3.0 Hz, 10-H), 7.16 (2H, d, *J* = 8.7 Hz, AA’-2H), 7.63 (2H, d, *J* = 9.0 Hz, BB’-2H), 7.84 (1H, s, C-H), 9.37 (1H, s, E-H), 9.53 (1H, s, F-H); ^13^C-NMR (75 MHz, CDCl_3_) *δ*: 176.32, 159.78, 143.30, 136.74, 134.31, 130.96, 129.30, 127.53, 127.22, 126.77, 117.25, 104.63, 100.58, 88.63, 81.25, 52.79, 44.61, 37.72, 36.63, 34.90, 31.21, 26.36, 24.92, 24.73, 20.62, 13.22.

*4-[(10S)-Dihydroartemisinin-10-oxy]benzaldehyde N^4^-(4-fluorophenyl)thiosemicarbazone* (**10b**). Yield 42.29%, m.p. 117–118 °C. MS (ESI) *m/z*: 556 (M+H); ^1^H-NMR (300 MHz, CDCl_3_) *δ*: 0.97 (3H, d, *J* = 6.0 Hz, 16-CH_3_), 1.03 (3H, d, *J* = 7.2 Hz, 15-CH_3_), 1.44 (3H, s, 14-CH_3_), 2.34–2.44 (1H, m, 4-H), 2.82-2.87 (1H, m, 9-H), 5.46 (1H, s, 12-H), 5.57 (1H, d, *J* = 3.0 Hz, 10-H), 7.16 (2H, d, *J* = 8.7 Hz, AA’-2H), 7.62 (2H, d, *J* = 9.0 Hz, BB’-2H), 7.85 (1H, s, C-H), 9.07 (1H, s, E-H), 9.68 (1H, s, F-H); ^13^C-NMR (75 MHz, CDCl_3_) *δ*: 175.82, 159.98, 143.54, 136.74, 131.76, 129.45, 129.17, 126.81, 126.11, 117.28, 104.64, 100.57, 88.64, 81.23, 52.78, 44.60, 37.73, 36.62, 34.90, 31.20, 26.35, 24.92, 24.74, 20.60, 13.20.

*4-[(10S)-Dihydroartemisinin-10-oxy]benzaldehyde N^4^-(4-ethoxyphenyl)thiosemicarbazone* (**10c**). Yield 31.80%, m.p. 114–116 °C. MS (ESI) *m/z*: 580 (M-H); ^1^H-NMR (300 MHz, CDCl_3_) *δ*: 0.96 (3H, d, *J* = 6.0 Hz, 16-CH_3_), 1.02 (3H, d, *J* = 7.2 Hz, 15-CH_3_), 1.44 (3H, s, 14-CH_3_), 2.33–2.44 (1H, m, 4-H), 2.81–2.86 (1H, m, 9-H), 4.05 (2H, q, *J* = 6.9 Hz, 4’-OCH_2_CH_3_), 5.46 (1H, s, 12-H), 5.55 (1H, d, *J* = 3.3 Hz, 10-H), 7.14 (2H, d, *J* = 8.7 Hz, AA’-2H), 7.60 (2H, d, *J* = 8.7 Hz, BB’-2H), 7.91 (1H, s, C-H), 9.02 (1H, s, E-H), 10.25 (1H, s, F-H); ^13^C-NMR (75 MHz, CDCl_3_) *δ*: 176.53, 159.79, 158.31, 143.24, 131.07, 129.34, 127.22, 117.22, 114.34, 104.62, 100.57, 88.62, 81.25, 55.77, 52.79, 44.62, 37.72, 36.63, 34.90, 31.21, 26.35, 24.92, 24.73, 20.60, 19.33, 13.20.

*4-[(10S)-Dihydroartemisinin-10-oxy]benzaldehyde N^4^-(2,5-dimethylphenyl)thiosemicarbazone* (**10d**). Yield 38.00%, m.p. 120–122 °C. MS (ESI) *m/z*: 566 (M+H); ^1^H-NMR (300 MHz, CDCl_3_) *δ*: 0.96 (3H, d, *J* = 5.7 Hz, 16-CH_3_), 1.02 (3H, d, *J* = 7.2 Hz, 15-CH_3_), 1.44 (3H, s, 14-CH_3_), 2.32 (3H, s, 2’-CH_3_), 2.37 (3H, s, 5’-CH_3_), 2.81–2.86 (1H, m, 9-H), 5.46 (1H, s, 12-H), 5.55 (1H, d, *J* = 3.3 Hz, 10-H), 7.14 (2H, d, *J* = 8.7 Hz, AA’-2H), 7.60 (2H, d, *J* = 8.7 Hz, BB’-2H), 7.90 (1H, s, C-H), 8.93 (1H, s, E-H), 10.13 (1H, s, F-H); ^13^C-NMR (75 MHz, CDCl_3_) *δ*: 176.91, 159.74, 143.29, 137.45, 134.48, 134.14, 131.69, 129.28, 127.64, 127.47, 127.29, 117.23, 104.62, 100.59, 88.62, 81.24, 52.79, 44.63, 37.72, 36.64, 34.92, 31.21, 26.36, 24.92, 24.74, 21.42, 20.61, 18.28, 13.21.

*4-[(10S)-Dihydroartemisinin-10-oxy]benzaldehyde N^4^-(3,5-dimethylphenyl)thiosemicarbazone* (**10e**). Yield 16.19%, m.p. 125–127 °C. MS (ESI) *m/z*: 566 (M+H); ^1^H-NMR (CDCl_3_) *δ*: 0.97 (3H, d, *J* = 5.7 Hz, 16-CH_3_), 1.03 (3H, d, *J* = 7.2 Hz, 15-CH_3_), 1.44 (3H, s, 14-CH_3_), 2.35 (6H, s, 3’-CH_3_, 5’-CH_3_), 2.81–2.86 (1H, m, 9-H), 5.46 (1H, s, 12-H), 5.56 (1H, d, *J* = 3.3 Hz, 10-H), 7.16 (2H, d, *J* = 8.7 Hz, AA’-2H), 7.61 (2H, d, *J* = 8.7 Hz, BB’-2H), 7.80 (1H, s, C-H), 9.08 (1H, s, E-H), 9.38 (1H, s, F-H); ^13^C-NMR (75 MHz, CDCl_3_) *δ*: 176.56, 159.77, 143.10, 137.43, 135.80, 135.15, 130.18, 129.32, 127.19, 126.46, 122.87, 117.23, 104.62, 100.58, 88.61, 81.24, 52.80, 44.62, 37.72, 36.64, 34.91, 31.21, 27.21, 26.35, 24.92, 24.73, 20.60, 19.68, 13.20. 

*4-[(10S)-Dihydroartemisinin-10-oxy]benzaldehyde N^4^-(3-chloro-2-methylphenyl)thiosemicarbazone* (**10f**). Yield 26.44%, m.p. 150–152 °C. MS (ESI) *m/z*: 584 (M-H); ^1^H-NMR (CDCl_3_) *δ*: 0.97 (3H, d, *J* = 6.0 Hz, 16-CH_3_), 1.03 (3H, d, *J* = 7.2 Hz, 15-CH_3_), 1.44 (3H, s, 14-CH_3_), 2.39 (3H, s, 2’-CH_3_), 2.81–2.86 (1H, m, 9-H), 5.46 (1H, s, 12-H), 5.56 (1H, d, *J* = 3.3 Hz, 10-H), 7.15 (2H, d, *J* = 8.7 Hz, AA’-2H), 7.61 (2H, d, *J* = 8.7 Hz, BB’-2H), 7.89 (1H, s, C-H), 8.95 (1H, s, E-H), 10.00 (1H, s, F-H); ^13^C-NMR (75 MHz, CDCl_3_) *δ*: 177.06, 159.74, 143.29, 138.08, 136.65, 133.88, 129.28, 127.32, 126.04, 117.22, 104.62, 100.58, 88.62, 81.25, 52.79, 44.62, 37.72, 36.64, 34.91, 31.21, 26.36, 24.93, 24.74, 20.84, 20.62, 14.33, 13.22.

*4-[(10S)-Dihydroartemisinin-10-oxy]benzaldehyde N^4^-(2-fluorophenyl)semicarbazone* (**10g**). Yield 72.28%, m.p. 175–177 °C. MS (ESI) *m/z*: 538 (M-H); ^1^H-NMR (CDCl_3_) *δ*: 0.97 (3H, d, *J* = 6.0 Hz, 16-CH_3_), 1.04 (3H, d, *J* = 7.5 Hz, 15-CH_3_), 1.45 (3H, s, 14-CH_3_), 2.34–2.45 (1H, m, 4-H), 2.81–2.86 (1H, m, 9-H), 5.48 (1H, s, 12-H), 5.56 (1H, d, *J* = 3.3 Hz, 10-H), 7.16 (2H, d, *J* = 8.7 Hz, AA’-2H), 7.61 (2H, d, *J* = 8.7 Hz, BB’-2H), 7.77 (1H, s, C-H), 8.49 (1H, s, E-H), 8.98 (1H, s, F-H); ^13^C-NMR (75 MHz, CDCl_3_) *δ*: 159.21, 154.37, 141.72, 136.54, 130.66, 128.57, 128.05, 128.00, 127.17, 124.02, 121.44, 117.25, 104.60, 100.69, 88.63, 81.26, 52.82, 44.66, 37.73, 36.65, 34.93, 31.25, 26.39, 24.94, 24.75, 20.63, 13.25.

*4-[(10S)-Dihydroartemisinin-10-oxy]benzaldehyde N^4^-(4-fluorophenyl)semicarbazone* (**10h**). Yield 44.63%, m.p. 124–126 °C. MS (ESI) *m/z*: 538 (M-H); ^1^H-NMR (CDCl_3_) *δ*: 0.97 (3H, d, *J* = 6.0 Hz, 16-CH_3_), 1.04 (3H, d, *J* = 7.5 Hz, 15-CH_3_), 1.45 (3H, s, 14-CH_3_), 2.34-2.45 (1H, m, 4-H), 2.82–2.87 (1H, m, 9-H), 5.48 (1H, s, 12-H), 5.56 (1H, d, *J* = 3.0 Hz, 10-H), 7.16 (2H, d, *J* = 8.7 Hz, AA’-2H), 7.59 (2H, d, *J* = 8.7 Hz, BB’-2H), 7.74 (1H, s, C-H), 8.08 (1H, s, E-H), 8.76 (1H, s, F-H); ^13^C-NMR (75 MHz, CDCl_3_) *δ*: 159.27, 154.12, 142.40, 136.95, 129.28, 128.78, 127.72, 121.17, 117.20, 104.61, 100.63, 88.62, 81.26, 52.81, 44.64, 37.73, 36.64, 34.92, 31.24, 26.37, 24.94, 24.74, 20.61, 13.23.

*4-[(10S)-Dihydroartemisinin-10-oxy]benzaldehyde N^4^-(4-ethoxyphenyl)semicarbazone* (**10i**). Yield 52.15%, m.p. 158–159 °C. MS (ESI) *m/z*: 564 (M-H); ^1^H-NMR (CDCl_3_) *δ*: 0.97 (3H, d, *J* = 5.7 Hz, 16-CH_3_), 1.04 (3H, d, *J* = 7.2 Hz, 15-CH_3_), 1.42 (3H, t, *J* = 7.2 Hz, 4’-OCH_2_CH_3_), 1.45 (3H, s, 14-CH_3_), 2.34–2.44 (1H, m, 4-H), 2.81–2.86 (1H, m, 9-H), 4.03 (2H, q, *J* = 6.9 Hz, 4’-OCH_2_CH_3_), 5.48 (1H, s, 12-H), 5.55 (1H, d, *J* = 3.3 Hz, 10-H), 7.15 (2H, d, *J* = 8.7 Hz, AA’-2H), 7.58 (2H, d, *J* = 8.7 Hz, BB’-2H), 7.73 (1H, s, C-H), 7.97 (1H, s, E-H), 8.81 (1H, s, F-H); ^13^C-NMR (75 MHz, CDCl_3_) *δ*: 159.18, 156.34, 154.57, 141.83, 131.32, 128.66, 128.03, 122.24, 117.15, 114.53, 104.58, 100.64, 88.60, 81.27, 55.82, 52.81, 44.66, 37.72, 36.65, 34.93, 31.25, 26.37, 24.93, 24.74, 20.61, 19.50, 13.23.

*4-[(10S)-Dihydroartemisinin-10-oxy]benzaldehyde N^4^-(2,5-dimethylphenyl)semicarbazone* (**10j**). Yield 80.05%, m.p. 172–173 °C. MS (ESI) *m/z*: 548 (M-H); ^1^H-NMR (CDCl_3_) *δ*: 0.97 (3H, d, *J* = 5.7 Hz, 16-CH_3_), 1.03 (3H, d, *J* = 7.2 Hz, 15-CH_3_), 1.45 (3H, s, 14-CH_3_), 2.33 (3H, s, 2’-CH_3_), 2.36 (3H, s, 5’-CH_3_), 2.81–2.86 (1H, m, 9-H), 5.48 (1H, s, 12-H), 5.55 (1H, d, *J* = 3.3 Hz, 10-H), 7.16 (2H, d, *J* = 8.7 Hz, AA’-2H), 7.57 (2H, d, *J* = 8.7 Hz, BB’-2H), 7.76 (1H, s, C-H), 8.12 (1H, s, E-H), 8.95 (1H, s, F-H); ^13^C-NMR (75 MHz, CDCl_3_) *δ*: 159.15, 154.62, 141.61, 133.83, 131.36, 128.57, 127.63, 122.14, 117.22, 104.59, 100.70, 88.62, 81.26, 52.83, 44.67, 37.72, 36.65, 34.95, 31.25, 26.38, 24.74, 21.12, 20.62, 18.34, 13.24.

*4-[(10S)-Dihydroartemisinin-10-oxy]benzaldehyde N^4^-(3,5-dimethylphenyl)semicarbazone* (**10k**). Yield 84.60%, m.p. 169–171 °C. MS (ESI) *m/z*: 548 (M-H); ^1^H-NMR (CDCl_3_) *δ*: 0.97 (3H, d, *J* = 6.0 Hz, 16-CH_3_), 1.04 (3H, d, *J* = 7.2 Hz, 15-CH_3_), 1.45 (3H, s, 14-CH_3_), 2.33 (6H, s, 3’-CH_3_, 5’-CH_3_), 2.81–2.86 (1H, m, 9-H), 5.48 (1H, s, 12-H), 5.56 (1H, d, *J* = 3.3 Hz, 10-H), 7.16 (2H, d, *J* = 8.7 Hz, AA’-2H), 7.59 (2H, d, *J* = 8.7 Hz, BB’-2H), 7.76 (1H, s, C-H), 8.05 (1H, s, E-H), 9.01 (1H, s, F-H); ^13^C-NMR (75 MHz, CDCl_3_) *δ*: 159.19, 154.33, 141.79, 137.44, 135.94, 132.01, 130.30, 128.67, 128.04, 121.58, 117.71, 117.17, 104.59, 100.67, 88.60, 81.27, 52.83, 44.67, 37.73, 36.66, 34.94, 31.26, 26.37, 24.94, 24.75, 20.61, 20.24, 19.03, 13.23.

*4-[(10S)-Dihydroartemisinin-10-oxy]benzaldehyde N^4^-(3-chloro-2-methylphenyl)semicarbazone* (**10l**). Yield 92.97%, m.p. 177–179 °C. MS (ESI) *m/z*: 568 (M-H); ^1^H-NMR (CDCl_3_) *δ*: 0.97 (3H, d, *J* = 5.7 Hz, 16-CH_3_), 1.03 (3H, d, *J* = 7.5 Hz, 15-CH_3_), 1.45 (3H, s, 14-CH_3_), 2.81–2.86 (1H, m, 9-H), 5.48 (1H, s, 12-H), 5.56 (1H, d, *J* = 3.3 Hz, 10-H), 7.57 (2H, d, *J* = 8.7 Hz, BB’-2H), 7.76 (1H, s, C-H), 8.20 (1H, s, E-H), 8.98 (1H, s, F-H); ^13^C-NMR (75 MHz, CDCl_3_) *δ*: 159.15, 154.79, 141.71, 137.49, 136.14, 128.56, 128.24, 126.43, 126.24, 120.86, 117.21, 104.59, 100.69, 88.62, 81.26, 52.83, 44.67, 37.73, 36.66, 34.94, 31.25, 26.38, 24.94, 24.74, 21.03, 20.62, 13.73, 13.24.

### 3.6. General Procedure for the Synthesis of 4-[(10S)dihydroartemisinin-10-oxycarbonyl]benzaldehyde N^4^-(substitued phenyl)semicarbazones ***12a-f***

To a CH_2_Cl_2_ solution (100 mL) of dihydroartemisinin (**2a**, 11.36 g, 40 mmol) and 4-formylbenzoic acid (7.21 g, 48 mmol), DCC (9.90 g, 48 mmol) and DMAP (1.47 g, 12 mmol) were added at 0–5 °C for 1 h. After 1 d reaction at room temperature, the mixture was filtered and filtrate was concentrated in vacuo. The residue was purified on silica gel column with petroleum ether/ethyl acetate (6:1) to afford **11**. Yield 40.46 %, m.p. 81–82 °C. MS (ESI) *m/z*: 439 (M+Na); ^1^H-NMR (CDCl_3_) *δ*: 0.94 (3H, d, *J* = 7.2 Hz, 16-CH_3_), 0.99 (3H, d, *J* = 5.7 Hz, 15-CH_3_), 1.43 (3H, s, 14-CH_3_), 2.35–2.45 (1H, m, 4-H), 2.74–2.81 1H, m, 9-H), 5.54 (1H, s, 12-H), 6.03 (1H, d, *J* = 9.6 Hz, 10-H), 7.97 (2H, d, *J* = 8.1 Hz, AA’-2H), 8.28 (2H, d, *J* = 8.1 Hz, BB’-2H), 10.12 (1H, s, C-H); ^13^C-NMR (75 MHz, CDCl_3_) *δ*: 190.89, 168.31, 162.30, 133.17, 117.52, 104.61, 100.73, 88.56, 81.13, 52.69, 44.62, 37.70, 36.63, 34.89, 31.10, 26.34, 24.72, 23.67, 20.60, 13.11. Equimolar quantities of **11** (2 mmol) and **7a-f** (2 mmol) was dissolved in EtOH (20 mL) catalyzed by glacial acetic acid (3 drops) and stirred for 2 h at room temperature. The precipitate was filtered and purified on silica gel column with CH_2_Cl_2_/CH_3_OH (80:1) to afford **12a-f**.

*4-[(10S)-Dihydroartemisinin-10-oxycarbonyl]benzaldehyde N^4^-(2-fluorophenyl)semicarbazone* (**12a**). Yield 17.62%, m.p. 145–147 °C. MS (ESI) *m/z*: 590 (M+Na); ^1^H-NMR (CDCl_3_) *δ*: 0.94 (3H, d, *J* = 7.2 Hz, 16-CH_3_), 0.99 (3H, d, *J* = 5.7 Hz, 15-CH_3_), 1.44 (3H, s, 14-CH_3_), 2.35–2.46 (1H, m, 4-H), 2.74–2.81 (1H, m, 9-H), 5.55 (1H, s, 12-H), 6.02 (1H, d, *J* = 9.9 Hz, 10-H), 7.72 (2H, d, *J* = 8.4 Hz, AA’-2H), 7.89 (1H, s, C-H), 8.14 (2H, d, *J* = 8.4 Hz, BB’-2H), 8.20 (1H, s, E-H), 9.46 (1H, s, F-H); ^13^C-NMR (75 MHz, CDCl_3_) *δ*: 166.32, 158.65, 154.63, 142.01, 136.62, 130.66, 128.63, 128.14, 128.03, 127.22, 124.03, 121.35, 117.33, 104.62, 100.70, 88.45, 81.27, 52.80, 44.51, 37.67, 36.73, 34.89, 31.20, 36.37, 24.90, 24.71, 20.59, 13.12.

*4-[(10S)-Dihydroartemisinin-10-oxycarbonyl]benzaldehyde N^4^-(4-fluorophenyl)semicarbazone* (**12b**). Yield 81.92%, m.p. 139–141 °C. MS (ESI) *m/z*: 590 (M+Na); ^1^H-NMR (CDCl_3_) *δ*: 0.93 (3H, d, *J* = 7.2 Hz, 16-CH_3_), 0.99 (3H, d, *J* = 5.7 Hz, 15-CH_3_), 1.43 (3H, s, 14-CH_3_), 2.35–2.45 (1H, m, 4-H), 2.73–2.81 (1H, m, 9-H), 5.55 (1H, s, 12-H), 6.02 (1H, d, *J* = 9.9 Hz, 10-H), 7.71 (2H, d, *J* = 8.4 Hz, AA’-2H), 7.91 (1H, s, C-H), 8.12 (2H, d, *J* = 8.1 Hz, BB’-2H), 8.13 (1H, s, E-H), 9.99 (1H, s, F-H); ^13^C-NMR (75 MHz, CDCl_3_) *δ*: 166.02, 159.32, 154.34, 142.36, 136.89, 129.33, 128.78, 128.91, 121.20, 117.15, 104.61, 100.60, 88.63, 81.32, 52.77, 44.56, 37.73, 36.55, 34.90, 31.22, 26.41, 24.91, 24.70, 20.62, 13.16.

*4-[(10S)-Dihydroartemisinin-10-oxycarbonyl]benzaldehyde N^4^-(4-ethoxyphenyl)semicarbazone* (**12c**). Yield 42.11%, m.p. 152–153 °C. MS (ESI) *m/z*: 616 (M+Na); ^1^H-NMR (CDCl_3_) *δ*: 0.93 (3H, d, *J* = 7.2 Hz, 16-CH_3_), 0.98 (3H, d, *J* = 5.1 Hz, 15-CH_3_), 1.43 (3H, s, 14-CH_3_), 2.35–2.45 (1H, m, 4-H), 2.73–2.80 (1H, m, 9-H), 4.04 (2H, q, *J* = 6.9 Hz, 4’-OCH_2_CH_3_), 5.54 (1H, s, 12-H), 6.01 (1H, d, *J* = 9.6 Hz, 10-H), 7.70 (2H, d, *J* = 8.1 Hz, AA’-2H), 7.90 (1H, s, C-H), 8.03 (1H, s, E-H), 8.12 (2H, d, *J* = 8.1 Hz, BB’-2H), 10.11 (1H, s, F-H); ^13^C-NMR (75 MHz, CDCl_3_) *δ*: 166.87, 159.52, 154.62, 142.78, 131.31, 128.70, 128.03, 122.17, 117.22, 114.51, 104.59, 100.60, 88.56, 81.32, 55.83, 52.79, 44.65, 37.71, 36.63, 34.90, 31.21, 26.36, 24.90, 24.72, 20.60, 19.45, 13.21.

*4-[(10S)-Dihydroartemisinin-10-oxycarbonyl]benzaldehyde N^4^-(2,5-dimethylphenyl)semicarbazone* (**12d**). Yield 85.69%, m.p. 155–156 °C. MS (ESI) *m/z*: 600 (M+Na); ^1^H-NMR (CDCl_3_) *δ*: 0.94 (3H, d, *J* = 7.2 Hz, 16-CH_3_), 0.99 (3H, d, *J* = 5.7 Hz, 15-CH_3_), 1.43 (3H, s, 14-CH_3_), 2.73–2.80 (1H, m, 9-H), 5.54 (1H, s, 12-H), 6.02 (1H, d, *J* = 9.6 Hz, 10-H), 7.67 (2H, d, *J* = 8.4 Hz, AA’-2H), 7.89 (1H, s, C-H), 8.11 (1H, s, E-H), 8.13 (2H, d, *J* = 8.4 Hz, BB’-2H), 9.68 (1H, s, F-H); ^13^C-NMR (75 MHz, CDCl_3_) *δ*: 167.14, 159.02, 154.61, 141.70, 134.23, 131.41, 128.48, 127.55, 121.11, 117.20, 104.62, 100.60, 88.56, 81.31, 52.78, 44.72, 37.66, 36.69, 34.90, 31.24, 26.42, 24.67, 21.10, 18.33, 13.21.

*4-[(10S)-Dihydroartemisinin-10-oxycarbonyl]benzaldehyde N^4^-(3,5-dimethylphenyl)semicarbazone* (**12e**). Yield 63.18%, m.p. 154–155 °C. MS (ESI) *m/z*: 600 (M+Na); ^1^H-NMR (CDCl_3_) *δ*: 0.93 (3H, d, *J* = 7.2 Hz, 16-CH_3_), 0.98 (3H, d, *J* = 5.7 Hz, 15-CH_3_), 1.43 (3H, s, 14-CH_3_), 2.73–2.80 (1H, m, 9-H), 5.54 (1H, s, 12-H), 6.01 (1H, d, *J* = 9.9 Hz, 10-H), 7.68 (2H, d, *J* = 8.4 Hz, AA’-2H), 7.93 (1H, s, C-H), 8.08 (1H, s, E-H), 8.11 (2H, d, *J* = 8.4 Hz, BB’-2H), 10.06 (1H, s, F-H); ^13^C-NMR (75 MHz, CDCl_3_) *δ*: 167.71, 159.22, 154.31, 141.77, 134.90, 132.01, 130.86, 128.66, 128.02, 121.56, 117.71, 117.20, 104.61, 100.68, 88.61, 81.32, 52.80, 44.73, 37.67, 36.70, 34.86, 31.20, 26.43, 24.91, 24.73, 20.60, 20.16, 19.21, 13.21.

*4-[(10S)-Dihydroartemisinin-10-oxycarbonyl]benzaldehyde N^4^-(3-chloro-2-methylphenyl)semicarbaz-one* (**12f**). Yield 63.54%, m.p. 173–174 °C. MS (ESI) *m/z*: 620 (M+Na); ^1^H-NMR (CDCl_3_) *δ*: 0.94 (3H, d, *J* = 6.9 Hz, 16-CH_3_), 0.98 (3H, d, *J* = 5.7 Hz, 15-CH_3_), 1.43 (3H, s, 14-CH_3_), 2.73–2.80 (1H, m, 9-H), 5.54 (1H, s, 12-H), 6.01 (1H, d, *J* = 9.9 Hz, 10-H), 7.67 (2H, d, *J* = 8.4 Hz, AA’-2H), 7.92 (1H, s, C-H), 8.12 (2H, d, *J* = 8.1 Hz, BB’-2H), 8.21 (1H, s, E-H), 10.19 (1H, s, F-H); ^13^C-NMR (75 MHz, CDCl_3_) *δ*: 166.32, 159.14, 154.81, 141.68, 137.11, 136.10, 128.61, 128.15, 126.42, 126.21, 120.94, 117.20, 104.56, 100.72, 88.61, 81.26, 52.80, 44.66, 37.70, 36.74, 34.92, 31.31, 26.42, 24.89, 24.70, 21.01, 20.64, 13.71, 13.20.

### 3.7. Inhibition Assays

Recombinant falcipain-2 (30 nM) was incubated for 30 min at room temperature in 100 mM NaOAc, pH 5.5, 10 mM dithiothreitol (DTT), with different concentrations of **10a-l** and **12a-f**. These solutions were prepared from stock in DMSO (maximum concentration of DMSO in the assay was 1%). After incubation, the substrate *Z*-Leu-Arg-AMC with the same buffer was added to a final concentration of 25 μM. The increase in fluorescence (excitation at 355 nm and emission at 460 nm) was monitored for 30 min at room temperature with an automated microplate spectrofluorimeter (Biotek, Synergy 2). IC_50_ values were determined from plots of percent activity over compound concentration using the GraphPad Prism software if inhibition rates were larger than 20% at 10 μM concentration level.

## 4. Conclusions

In conclusion, a total of 18 dihydroartemisinin derivatives, **10a-l** and **12a-f**, were synthesized from the natural product artemisinin (**1**) and anilines through simplified procedures. All target compounds were obtained as single isomers as confirmed by ^1^H-NMR through the key intermediates **9** and **11**. The biological evaluation and molecular docking studies indicated that this new class of dihydroartemisinin derivatives could be identified as potential falcipain-2 inhibitors and the preliminary SARs could serve as a foundation for further investigation of antimalarial drugs.

## Figures and Tables

*Reagents and conditions*: (a) CS_2_, NH_3_⋅H_2_O, EtOH, rt, 1 h; (b) ClCH_2_COONa, rt, 30 min; (c) NH_2_NH_2_⋅H_2_O, rt, 1 h; (d) phenyl chloroformate, pyridine, CH_2_Cl_2_, ice-water bath, rt, 4 h; (e) NH_2_NH_2_⋅H_2_O, reflux, 12 h; (f) NaBH_4_, CH_3_OH, 0–5 °C, 3 h; (g) TFAA, Et_3_N, CH_2_Cl_2_, −5–0 °C, 1 d; (h) 4-hydroxybenzaldehyde, −5–0 °C, 12 h; (i) **5a-f** or **7a-f**, CH_3_COOH, EtOH, rt, 2 h; (j) 4-formylbenzoic acid, DCC, DMAP, ice-water bath, rt, 1 d; (k) **7a-f**, CH_3_COOH, EtOH, rt, 2 h.

**Table 1:** Inhibition activity of compounds **10a-l** and **12a-f** against *P. falciparum* falcipain-2 *in vitro*.

Compd.	X	R_1_	R_2_	Inhibition rate^a^ (%)	IC_50_^b^ (μM)
**10a**	S	H	2-F	72.48	2.51
**10b**	S	H	4-F	59.76	2.17
**10c**	S	H	4-OCH_2_CH_3_	87.42	0.52
**10d**	S	2-CH_3_	5-CH_3_	75.28	0.55
**10e**	S	3-CH_3_	5-CH_3_	74.06	1.35
**10f**	S	3-Cl	2-CH_3_	75.62	0.984
**10g**	O	H	2-F	76.74	2.25
**10h**	O	H	4-F	84.60	1.02
**10i**	O	H	4-OCH_2_CH_3_	72.70	0.7
**10j**	O	2-CH_3_	5-CH_3_	59.01	2.28
**10k**	O	3-CH_3_	5-CH_3_	87.88	1.03
**10l**	O	3-Cl	2-CH_3_	81.82	2.54
**12a**	O	H	2-F	79.32	0.47
**12b**	O	H	4-F	69.25	0.65
**12c**	O	H	4-OCH_2_CH_3_	75.89	0.29
**12d**	O	2-CH_3_	5-CH_3_	76.61	2.96
**12e**	O	3-CH_3_	5-CH_3_	71.62	0.57
**12f**	O	3-Cl	2-CH_3_	74.67	10.63
E-64				100.0	0.0197
DMSO				0	

^a^ Inhibition rates were evaluated at 10 μM concentration level; ^b^ IC_50_ values were determined if above inhibition rates were larger than 20%.

## References

[1] Begue J.P., Bonnet D.D. (2005). The future of antimalarials: Artemisinin and synthetic endoperoxides. Drugs Future.

[2] Klayman D.L. (1985). Qinghaosu (artemisinin): An antimalarial drug from China. Science.

[3] World Health Organization (WHO) (2010). Global Report on Antimalarial Efficacy and Drug Resistance: 2000-2010.

[4] World Health Organization (WHO) (2011). Global Plan for Artemisinin Resistance Containment (GPARC).

[5] Ettari R., Bova F., Zappala M., Grasso S., Micale N. (2010). Falcipain-2 inhibitors. Med. Res. Rev..

[6] Rosenthal P.J., Sijwali P.S., Singh A., Shenai B.R. (2002). Cysteine proteases of malaria parasites: Targets for chemotherapy. Curr. Pharm. Des..

[7] Klayman D.L., Bartosevich J.F., Griffin T.S., Mason C.J., Scovill J.P. (1979). 2-Acetylpyridine thiosemicarbazones. 1. A new class of potential antimalarial agents. J. Med. Chem..

[8] Chipeleme A., Gut J., Rosenthal P.J., Chibale K. (2007). Synthesis and biological evaluation of phenolic Mannich bases of benzaldehyde and (thio)semicarbazone derivatives against the cystein protease falcipain-2 and a chloroquine resistant strain of Plasmodium falciparum. Bioorg. Med. Chem..

[9] Walcourt A., Loyevsky M., Lovejoy D.B., Gordeuk V.R., Richardson D.R. (2004). Novel aroylhydrazone and thiosemicarbazone iron chelators with anti-malarial activity against chloroquine-resistant and -sensitive parasites. Int. J. Biochem. Cell Biol..

[10] Liang J., Li Y. (1996). Synthesis of the aryl ether derivatives of artemisinin. Chin. J. Med. Chem..

[11] Brossi A., Venugopalan B., Dominguez G.L., Yeh H.J.C., Flippen A.J.L., Buchs P., Luo X.D., Milhous W., Peters W. (1988). Arteether, a new antimalarial drug: Synthesis and antimalarial properties. J. Med. Chem..

[12] Li Y., Yu P.L., Chen Y.X., Ji R.Y. (1982). Study on analogs of artemisinin II. The synthesis of some carboxylic esters and carbonates of dihydroarteannuin by using 4-(*N*,*N*-dimethylamino)pyridine as an active acylation catalyst. Acta Chim. Sinica.

[13] Dimri A.K., Parmar S.S. (1978). Synthesis of 3-aryl-4-oxothiazolin-2-yl-(4-ethoxy-3-methoxy)phenyl hydrazones as possible anticonvulsants. J. Heterocycl. Chem..

[14] Thirumurugan R., Sriram D., Saxena A., Stables J., Yogeeswari P. (2006). 2,4-Dimethoxyphenylsemicarbazones with anticonvulsant activity against three animal models of seizures: Synthesis and pharmacological evaluation. Bioorg. Med. Chem..

[15] O’Neil P.M., Miller A., Bishop L.P.D., Hindley S., Maggs J.L., Ward S.A., Roberts S.M., Scheinmann F., Stachulski A.K., Posner G.H., Park B.K. (2001). Synthesis, antimalarial activity, biomimetic iron(II) chemistry, and *in vivo* metabolism of novel, potent C-10-phenoxy derivatives of dihydroartemisinin. J. Med. Chem..

[16] Yang Z.S., Zhou W.L., Sui Y., Wang J.X., Wu J.M., Zhou Y., Zhang Y., He P.L., Han J.Y., Tang W., Li Y., Zuo J.P. (2005). Synthesis and immunosuppressive activity of new artemisinin derivatives. Part I. [12(*β* or *α*)-dihydroartemisininoxy]phen(ox)yl aliphatic acids/esters. J. Med. Chem..

[17] Shenai B.R., Sijwali P.S., Singh A., Rosenthal P.J. (2000). Characterization of native and recombinant falcipain-2, a principal trophozoite cysteine protease and essential hemoglobinase of *plasmodium falciparum*. J. Biol. Chem..

[18] Morris G.M., Goodsell D.S., Halliday R.S., Huey R., Hart W.E., Belew R.K., Olson A.J. (1998). Automated docking using a Lamarckian genetic algorithm and an empirical binding free energy function. J. Comput. Chem..

